# From severity scoring to predictive analytics: the emerging role of AI in neurosurgery

**DOI:** 10.3389/fneur.2026.1753844

**Published:** 2026-06-26

**Authors:** Yongyi Huang

**Affiliations:** Department of Neurosurgery (Neurocritical Care), Wuzhou Red Cross Hospital, Wuzhou, Guangxi, China

**Keywords:** artificial intelligence, augmented surgery, deep learning, neurosurgery, predictive analytics

## Abstract

The rapid integration of artificial intelligence (AI) into neurosurgical practice is transforming every phase of patient care from diagnostic imaging and preoperative planning to intraoperative decision-making and postoperative management. This narrative review traces the evolution of data-driven neurosurgery, beginning with traditional severity scoring systems and advancing toward predictive analytics and intelligent automation. By examining structured data (such as electronic health records and laboratory values) alongside complex unstructured inputs (including neuroimaging, surgical videos, and free-text notes), can extract clinically meaningful patterns, with reported performance metrics such as Dice scores of 0.82–0.84 for tumor segmentation and AUC values of 0.80–0.90 for molecular prediction and outcome forecasting. Applications in lesion detection, surgical navigation, prognostication, and rehabilitation are discussed, along with critical challenges in interpretability, data harmonization, bias mitigation, and regulatory approval. Emerging paradigms such as federated learning, generative AI, and continuous learning ecosystems are also explored as future pathways toward ethical, adaptive, and globally connected neurosurgical intelligence. As a narrative review, this work synthesizes key developments qualitatively; specific performance metrics and limitations regarding systematic selection, quantitative synthesis, and variable model validation are addressed. Ultimately, AI serves not as a replacement for the neurosurgeon but as a cognitive collaborator, augmenting precision, efficiency, and patient-centered outcomes in modern neurosurgery.

## 1 Introduction

Neurosurgery is currently experiencing a significant evolution, driven by the urgent need for greater precision and predictability in managing complex medical conditions, including traumatic brain injuries and intracranial tumors (1). For many years, the field has utilized standardized severity scoring systems to help guide clinical decision-making and prognostication. These scores, such as commonly known Glasgow Coma Scale (GCS) and more detailed tools like the APACHE II or disease-specific outcome scales, have provided an essential framework for assessing risk and predicting long-term patient outcomes (2, 3). They represent the initial efforts to simplify complex patient profiles into measurable data, thereby creating a standardized method for evaluating the severity of injuries.

However, as the landscape of neurosurgery evolves, it faces challenges due to the influx of high-dimensional data derived from various sources, such as advanced imaging techniques, continuous electrophysiological monitoring, genomic sequencing, and comprehensive electronic health records (4, 5). This complexity highlights the shortcomings of traditional statistical approaches such as logistic regression, which, while useful for analyzing a limited set of variables, often struggle to account for the intricate, non-linear interactions that characterize medical conditions and recovery processes (6). Their dependence on manual feature selection and assumptions about data structures limits their effectiveness in uncovering the predictive insights present in modern clinical datasets.

In this context, artificial intelligence (AI) is emerging as a transformative force. The intersection of Artificial Intelligence and the clinical setting is considerably increasing nowadays (7). AI methods can facilitate quick and thorough analysis of the extensive clinical data produced in contemporary healthcare environments, reaching levels of insight that are unattainable by human analysis (8). Through the enhancement of diagnostic accuracy, the streamlining of workflows, and real-time data analysis, AI technologies ultimately lead to improved patient outcomes (9). Moreover, AI-guided guidelines may be helpful in decision-making capacity of clinicians in medical applications and presents opportunities for enhancing personalized treatment strategies within the intricate field of neuroendovascular procedures (10, 11).

Understanding the different components of the AI landscape including Machine Learning (ML), Deep Learning (DL), and predictive analytics is crucial to recognizing its potential impact (12). Machine learning algorithms autonomously identify data patterns without explicit programming, while deep learning a subset that employs complex neural networks—excels in processing unstructured data like medical images and videos. These technologies underpin advanced predictive analytics, shifting from static severity scoring to dynamic, personalized prognostic insights (13).

**Figure 1:**
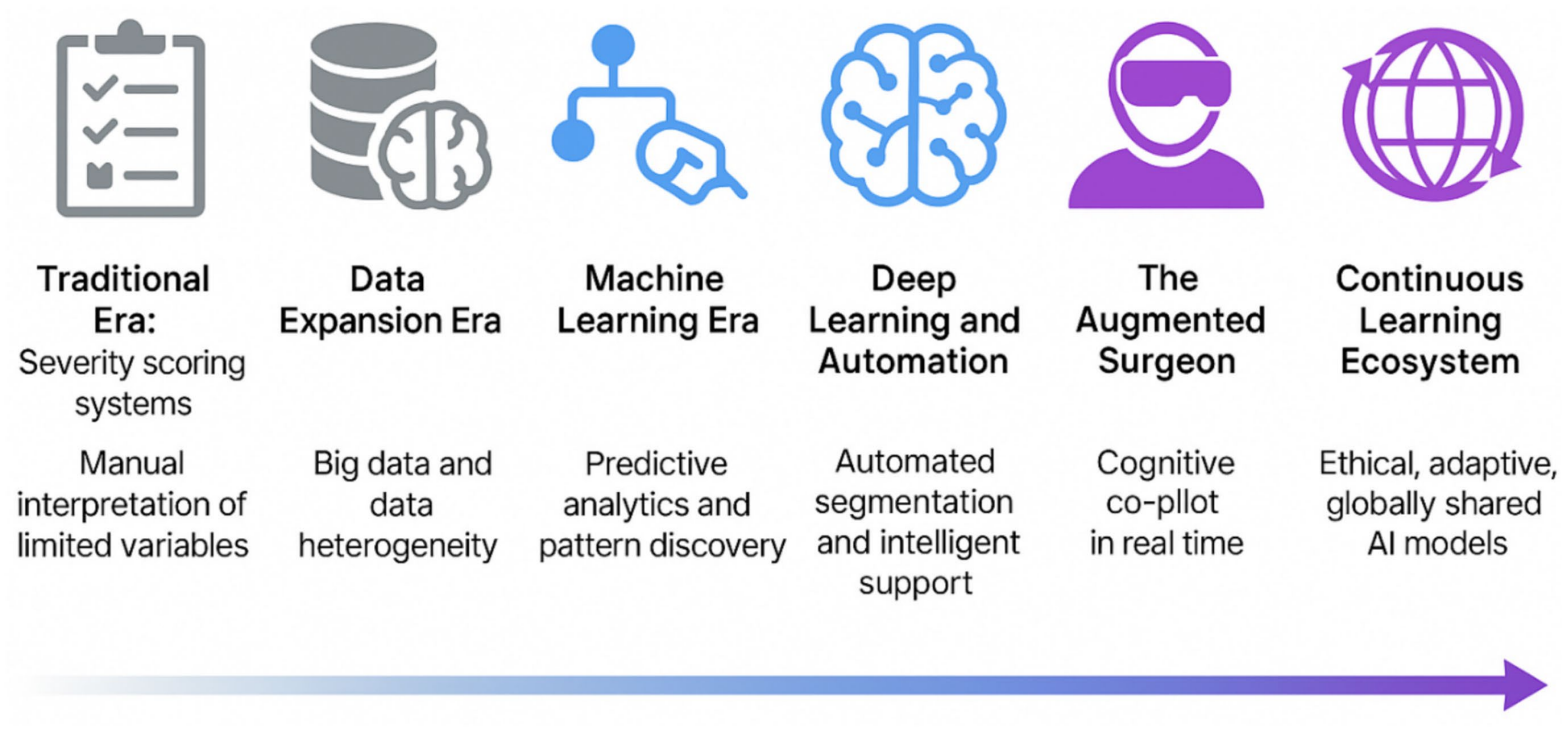
Conceptual overview of the evolution of data-driven neurosurgery. The field has progressed from traditional, static severity scoring systems based on limited clinical parameters toward dynamic, multi-modal, and continuously learning artificial intelligence (AI) frameworks. Integrating structured and unstructured data sources including electronic health records, imaging, biosignals, and surgical videos AI supports neurosurgeons in diagnosis, prognostication, intraoperative guidance, and personalized postoperative care, culminating in the vision of an augmented, adaptive neurosurgical ecosystem.

This narrative review aims to critically evaluate the growing role of AI throughout the entire spectrum of neurosurgical care. It will explore applications of AI in enhancing diagnostic accuracy, improving preoperative planning, facilitating intelligent prognostication, providing intraoperative assistance, and enabling personalized postoperative management. Additionally, this review will address significant challenges faced in the clinical implementation of AI, including issues related to model interpretability, data privacy, and algorithmic bias, while also discussing future directions for the responsible incorporation of AI into neurosurgical practices. By synthesizing existing evidence and fostering critical discourse, this review intends to provide a thorough overview of how AI stands to enhance the capabilities of neurosurgeons, paving the way for new era of precision neurosurgery. The conceptual evolution from traditional scoring systems to AI-driven precision neurosurgery is illustrated in Figure 1.

### 1.1 Scope and methodology of this narrative review

This article is a narrative review that aims to provide a broad, integrative overview of the evolution and applications of AI in neurosurgery rather than a systematic review with exhaustive literature retrieval and quantitative synthesis. We conducted a targeted search of PubMed, Google Scholar, and Web of Science (up to March 2025) using combinations of terms such as “artificial intelligence,” “machine learning,” “deep learning,” “neurosurgery,” “neuro-oncology,” “radiomics,” “intraoperative,” and specific applications (e.g., “tumor segmentation,” “outcome prediction”). References were selected based on relevance, impact, recency, and representation of key concepts, prioritizing high-quality original research, systematic reviews, and expert consensus where available. Where available, we prioritized studies reporting key performance metrics (e.g., Dice similarity coefficient, area under the ROC curve [AUC], sensitivity/specificity, F1-score) and external validation. Because no formal PRISMA protocol, predefined eligibility criteria, or risk-of-bias assessment across all studies was applied, this approach is susceptible to selection bias (e.g., preferential inclusion of positive or English-language studies) and limits the generalizability of conclusions. Furthermore, the heterogeneous nature of the included studies (varying methodologies, sample sizes, and outcome measures) precluded a quantitative meta-analysis. These limitations are discussed in detail in Section 7. Despite these constraints, the narrative format allows for critical synthesis of conceptual advances, clinical translation pathways, and forward-looking perspectives that are particularly timely given the rapid pace of AI development in neurosurgery.

## 2 The foundation: data types and preprocessing for AI in neurosurgery

The effectiveness of any AI model is fundamentally tied to the quality, quantity, and diversity of the data used for its training (14). In the context of neurosurgery, the data landscape is particularly rich and varied, offering significant opportunities along with notable foundational challenge (15). Understanding this data is essential for clinically applicable AI tools by neurosurgeons.

### 2.1 Structured data: electronic health records (EHRs), laboratory values, and demographics

Structured data forms the foundation of many predictive models in neurosurgery (16). This type of data is organized into predefined formats, such as rows and columns in a database, and include demographic information (age, gender), vital signs, laboratory values, medication records, and coded diagnosis or procedures obtained from EHRs (17). Such structured data can be effectively analyzed using traditional statistical methods and machine learning algorithms, including logistic regression and support vector machine (18). For example, preoperative structured data may be used to assess the risk of postoperative complications, such as venous thromboembolism or surgical site infection. However, the utility of this data can be limited by issues such as inconsistencies, missing entries, and the pitfalls of reductionism, which may overlook important aspects of clinical setting (19).

### 2.2 Unstructured and complex data: neuroimaging, operative videos, and free-text notes

Recent advancements in neurosurgical AI are increasingly driven by the analysis of unstructured and complex data, which constitutes a significant portion of the clinical record (20). This includes neuroimaging, Operative Videos, and Free-Text Notes (21, 22). Neuroimaging techniques like MRI, CT, and DSA provide comprehensive sub-visual information. Deep learning methods, especially Convolutional Neural Networks (CNNs), enable automatic tumor segmentation, aneurysm identification, peritumoral edema quantification, and extraction of radiomic features beyond human visual detection (23, 24). These features can correlate with genomic markers and patient outcomes. Secondly, operating videos of surgical procedures offers a unique opportunity for objective skill assessment, phase recognition in complex surgeries, and the development of context-aware intraoperative guidance systems (25). AI can assist in identifying critical anatomical structures, tracking instruments, and signaling potential deviations from established surgical techniques (26). Finally, operative reports, discharge summaries, and clinical notes contain a wealth of qualitative information. Natural Language Processing (NLP) techniques can be employed to extract critical insights, such as the extent of surgical resection or neurological examination findings, that would otherwise remain in an unstructured format (27, 28). As summarized in Table 1, various structured and unstructured data sources contribute to the development of AI systems in neurosurgery, each presenting unique analytical opportunities and challenges.

**Table 1:** Data types utilized for AI development in neurosurgery.

Data type	Examples	Common AI methods	Applications	Challenges/ limitations
Structured data	EHRs, demographics, lab values, vital signs	Logistic regression, Random Forest, Gradient Boosting	Predict postoperative complications, stratify risk	Missing data, reductionism, inter-institutional variability
Unstructured imaging data	MRI, CT, DSA	CNNs, Autoencoders, Radiomics	Tumor segmentation, aneurysm detection, radiogenomic prediction	Large labeled datasets, imaging heterogeneity
Operative videos	Microsurgical or endoscopic videos	Computer Vision, Temporal CNNs	Phase recognition, skill assessment	High labeling burden, privacy concerns
Textual data	Operative reports, notes, discharge summaries	NLP, Transformers (BERT, GPT)	Information extraction, outcome prediction	Ambiguous language, lack of standardization
Physiologic/Biosignal data	EEG, ECoG, ICP, vital-sign streams	RNNs, LSTM, Time-series learning	Detect deterioration, seizure forecasting	Noise, artifact handling, data volume

### 2.3 The crucial challenge: data curation, labeling, and harmonization

Before any algorithm can learn, the data must undergo meticulous curation. Neurosurgical data often exists in silos across various hospital systems and imaging archives with incompatible formats. The harmonization of MRI sequences from different standardization across institutions is a complex yet necessary task for creating large, usable data (29, 30). Moreover, supervised learning, the predominant paradigm in AI, requires that data be labeled by expert professionals (31). This may involve radiologists delineating tumor boundaries on multiple MRI slices or senior neurosurgeons annotating pivotal steps in surgical videos. This labeling process is resource-intensive and can introduce variability, thus generating noise within the training data (32).

### 2.4 Ethical and privacy considerations in neurosurgical data repositories

The consolidation of sensitive patient data into large repositories raises significant ethical and privacy concerns (33). Given the nature of neurosurgical conditions which often lead to profound life changes, this data is highly confidential. At first, the use of patient data for research purposes, especially when employed to train algorithms for future commercial applications, necessitates transparent and thorough informed consent processes that traditional protocols may not adequately address (34). Furthermore, while careful measures can be taken to anonymize data, the risk of re-identification remains a concern (35, 36). Proper safeguards must be in place to protect patient privacy in the development and utilization of AI tools.

## 3 AI in diagnostic precision and preoperative planning

**Figure 2:**
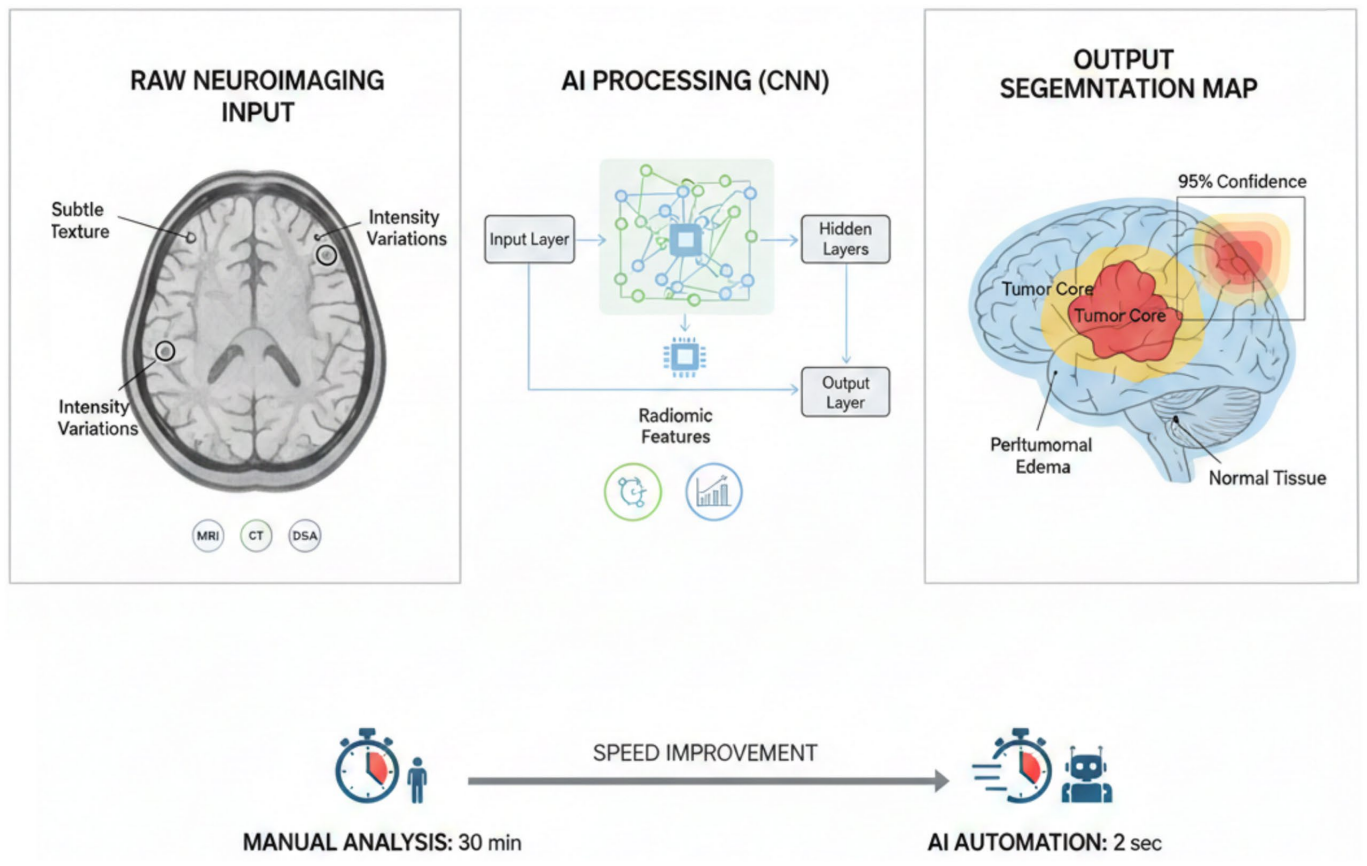
AI-driven lesion detection and segmentation in neuroimaging.

The emergence of AI, particularly through advancements in deep learning and radiomics, is significantly transforming the field of neurosurgery (37). Moving past traditional imaging techniques, AI algorithms enhance the capabilities of neurosurgeons by delivering unparalleled diagnostic accuracy and enabling sophisticated, personalized preoperative planning (38). AI modalities have illustrated potential in lesion detection, image-based phenotypic and genotypic analysis, and the development of optimal surgical pathways (Figure 2) (39).

A multi-panel workflow illustrating how artificial intelligence enhances diagnostic precision and preoperative planning in neurosurgical imaging. The left panel shows raw MRI/CT inputs with subtle intensity and texture cues used by AI models. The central panel depicts a convolutional neural network architecture extracting radiomic features such as shape descriptors and intensity histograms. The right panel presents automated output, including segmented tumor boundaries, peritumoral edema, probability heatmaps, and modality-specific annotations (MRI, CT, DSA). A bottom timeline highlights the improvement in processing speed (e.g., conventional manual review versus AI-based inference). This figure demonstrates the transition from manual interpretation to AI-assisted, high-precision radiogenomic analysis.

### 3.1 Automated lesion detection and segmentation: brain tumors, aneurysms, and hematomas

The first step in any neurosurgical procedure is the precise identification of the pathologic structure (40). CNNs have excelled in automatically detecting and segmenting critical lesions in brain imaging, such as MRI and CT scans. Recent meta-analyses report pooled Dice scores of approximately 0.82–0.84 (95% CI: 0.80–0.87) for glioma segmentation on multi-modal MRI, with higher performance for whole tumor (often >0.85) than enhancing tumor or edema subregions (41, 42). For instance, in the case of brain tumors, including gliomas and meningiomas, AI models can accurately segment the tumor core, the surrounding edema, and normal tissues at levels comparable to, and in some cases exceeding, those of expert manual delineation (43, 44). This automation minimizes variability between different observers and significantly accelerates the diagnostic workflow, which is vital in time-sensitive medical environments. However, performance drops on external datasets due to imaging heterogeneity, and Hausdorff distances (measuring boundary errors) often remain clinically relevant (typically 2–10 mm), underscoring the need for expert oversight (42). In addition to tumor detection, AI has proven incredibly useful in vascular neurosurgery. Algorithms can analyze extensive datasets of magnetic resonance angiography (MRA) or CT Angiography (CTA) to identify potential intracranial aneurysms, including those that are small or have atypical shapes that might not be detected by human specialists (45). Additionally, in cases of traumatic brain injury and hemorrhagic stroke, AI systems can quickly identify and quantify intracranial hematomas on non-contrast CT scans, offering essential decision support for emergency surgical interventions (46, 47). This level of automated segmentation establishes a reliable, quantitative baseline for tracking disease progression and informing surgical planning.

### 3.2 Radiomics and radiogenomics: decoding tumor phenotype and genotype from images

Radiomics entails the extraction of numerous quantitative features from medical images that might not be visible to the naked eye (48). AI models are subsequently used to analyze and interpret these complex datasets in order to predict tumor characteristics. Radiogenomics builds on this by correlating these radiomic features with the tumor’s underlying genetic makeup (39). In the field of neuro-oncology, this innovative approach is reshaping preoperative assessments. AI models have the capability to non-invasively predict the mutational status of crucial genes such as IDH1, MGMT promoter methylation, and the 1p/19q co-deletion in gliomas, using only preoperative MRIs (49, 50). With pooled AUC values around 0.85 (sensitivity ~79%, specificity ~80%) across meta-analyses of radiomics/ML models. Deep learning approaches sometimes achieve higher AUCs (0.90 + in internal validation) but show greater variability on external testing (51). These predictive performances offer encouraging non-invasive insights but are currently limited by retrospective designs, lack of prospective validation, and dependence on manual segmentation in many pipelines. This predictive information provides critical prognostic insights and aids in selecting appropriate neoadjuvant therapies, potentially reducing the need for invasive biopsy and genomic analysis. Radiomics signatures can differentiate between high-grade and low-grade gliomas, distinguish glioblastoma from primary central nervous system lymphoma, and forecast early recurrence risks, influencing the aggressiveness of surgical interventions (52, 53).

### 3.3 Surgical trajectory planning: enhancing safety and efficacy with AI-generated models

Effective preoperative planning is essential for achieving maximal tumor resection while minimizing patient morbidity (54). AI contributes significantly to this planning process by generating optimized surgical trajectories. By synthesizing multiple imaging modalities, such as structural MRI, Diffusion tensor imaging (DTI) to map white matter tracts, and functional MRI, AI algorithms can construct patient-specific 3D models of the surgical landscape. These models serve as a platform for simulating various surgical corridors (55).

Advanced path-planning algorithms, similar to those found in robotics, can automatically propose trajectories that bypass critical brain areas, such as the primary motor cortex or essential language pathways, while minimizing interference with major blood vessels (56). In the context of functional neurosurgery, especially procedures like deep brain stimulation (DBS), AI aids in accurately targeting subcortical nuclei while avoiding vascular structures, thereby improving both safety and therapeutic outcomes (57). This computational methodology offers a quantitative, reproducible foundation for surgical planning, reducing dependence on purely heuristic, experience-based choices.

## 4 Intelligent prognostication: from admission to long-term outcomes

**Figure 3:**
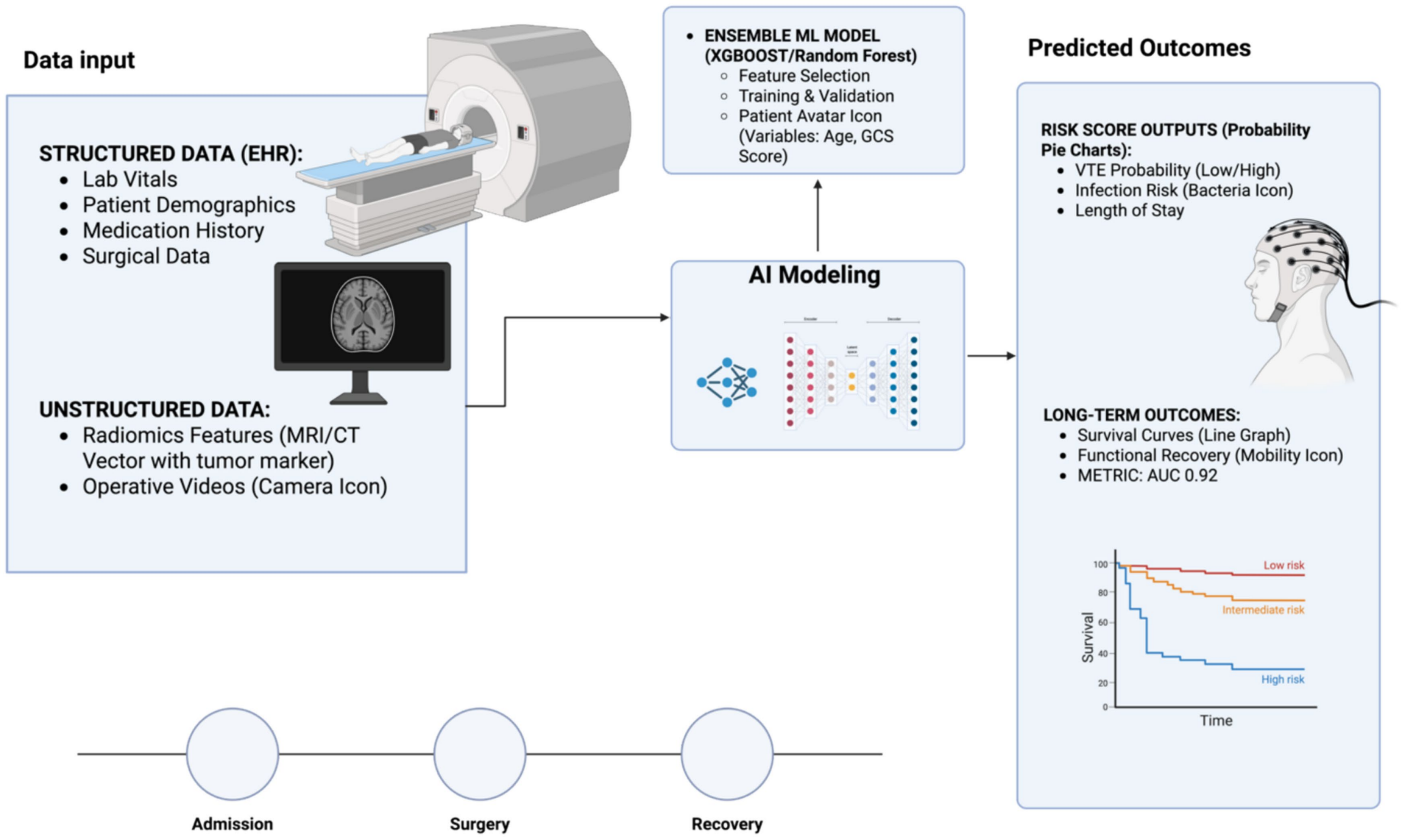
Predictive analytics workflow for postoperative complications.

Accurate prediction of patient outcomes is a fundamental component of high-quality surgical care, enabling personalized treatment strategies, establishing realistic expectations, and optimizing resource allocation (58). The integration of AI is significantly enhancing this prognostic capability within the field of neurosurgery. By synthesizing complex, multi-modal data, ranging from demographic and clinical variables to high-dimensional imaging and genomic information, AI models are outperforming traditional risk assessment methods to deliver dynamic, individualized prediction from the point of admission through to long-term recovery (Figure 3) (59).

### 4.1 Predicting postoperative complications: infections, venous thromboembolism, and delirium

Postoperative complications can have a substantial impact on patient morbidity, duration of hospitalization, and overall healthcare costs. AI models, substantially those that utilize electronic health record (EHR) data, are demonstrating considerable efficacy in identifying patients at higher risk of specific adverse events. For instance, concerning surgical site infection (SSI), machine learning algorithms can incorporate preoperative and interoperative factors- including body mass index, operation duration, ASA score, and preoperative glucose levels- to generate patient-specific risk scores that present superior discriminative ability compared to conventional logistic regression models (60, 61). In the context of venous thromboembolism (VTE), a common and potentially severe complication among neurosurgical patients, AI can adeptly assess influential factors such as patient mobility, cancer diagnosis, and the nature of surgical procedures to identify individuals at high risk, thereby facilitating timely prophylactic measures (62). Moreover, AI is also being employed to predict more subtle complications, such as postoperative delirium. By evaluating preoperative cognitive assessments, medication history, and laboratory results, predictive models can alert clinicians to patients who may benefit from tailored non-pharmacological delirium prevention protocol (63, 64). This proactive approach, enabled by the ability of AI to identify intricate patterns across diverse data sources, represents a significant shift from reactive management to preemptive risk mitigation.

AI models have reported AUCs of 0.75–0.88 for surgical site infections and venous thromboembolism prediction, often outperforming traditional scores in retrospective cohorts. For postoperative delirium, models using EHR data achieve AUCs ~0.70–0.85. These results are promising for risk stratification, yet most lack prospective, multi-center validation, and calibration (agreement between predicted and observed risks) is infrequently reported (65).

### 4.2 Survival analysis in neuro-oncology and neuro-trauma

Accurate survival prediction is essential for counseling patients and their families, as well as for making complicated decisions regarding treatment aggressiveness. In the setting of neuro-oncology, AI has progressed beyond simplistic histopathological grading (1). By integrating clinical data with imaging features, and molecular markers, AI models can offer highly individualized survival predictions (66). For example, in glioblastoma cases, algorithms that incorporate age, Karnofsky Performance Scale Index, extent of surgical resection, and radiomic signatures derived from perioperative MRIs have demonstrated a superior ability to categorize patients into distinct survival subgroups compared to models that rely solely on clinical data (67). Digital biomarkers reflect intratumoral heterogeneity and biological aggression not fully captured by standard imaging. In neurotrauma, predicting mortality and long-term outcomes after traumatic brain injury is crucial. AI models utilizing admission GCS, pupil reactivity, CT findings, and biomarkers like S100B demonstrate high accuracy in forecasting mortality and adverse outcomes. These models offer clinicians quantitative, evidence-based prognostic estimates, aiding surgical decisions and enhancing communication with surrogates.

While AI-driven survival prediction models in glioma have demonstrated promising discriminative performance, their clinical applicability remains limited by methodological constraints. A recent retrospective study from Città della Salute e della Scienza-Molinette University Hospital developed the DAK-75 score to predict 12-month mortality in patients aged ≥75 years with high-grade gliomas. Based on clinical presentation, tumor location, and KPS, the model showed good performance (AUROC 0.822) and stratified patients into risk groups, with higher scores associated with increased mortality and lower likelihood of surgical intervention. However, its retrospective single-center design and lack of external validation limit immediate clinical applicability, underscoring the need for prospective, multi-center validation and integration with advanced AI-based models (68). However, these models often rely on retrospective datasets with limited external validation and insufficient calibration assessment, raising concerns about their generalizability across diverse patient populations. Importantly, many models prioritize performance metrics such as AUC without adequately addressing clinical interpretability, decision thresholds, and real-world integration into treatment planning.

### 4.3 Forecasting functional and neurological recovery

For many neurosurgical interventions, the ultimate objective extends beyond mere survival to encompass the restoration of functional capabilities (69). AI is increasingly being utilized to predict trajectories of neurological recovery. In cases of spinal cord injury, for example, machine leaning models that combine initial International Standards for Neurological Classification of SCI (ISNCSCI) scores with DTI parameters have shown substantial accuracy in predicting ling-term motor and sensory outcomes (70, 71). This predictive capacity facilitates the early identification of patients who may be suitable candidates for aggressive rehabilitation or innovative neurodegenerative treatments. In the field of cerebrovascular neurosurgery, particularly following subarachnoid hemorrhage or ischemic stroke, AI models can evaluate the probability of cognitive deficit or chronic disability (72, 73). By analyzing factors such as the severity of initial hemorrhage, subsequent development of vasospasm, and perioperative perfusion data, these models assist in tailoring long-term rehabilitation plans and establishing appropriate recovery goals. This focus on quality of life aligns with the principles of the value-based healthcare, moving beyond survival outcome alone.

In vascular neurosurgery and cerebrovascular pathology, machine learning models are increasingly used to predict outcomes such as functional recovery, complication risk, and survival. Aneurysmal subarachnoid hemorrhage (aSAH) due to anterior communicating artery (AcoA) aneurysms is associated with high mortality and disability, with rebleeding being a critical determinant of poor outcome. In a retrospective cohort of 50 patients undergoing microsurgical clipping, machine learning approaches (MRMR feature selection and LASSO modeling) identified key prognostic variables (74). Preoperative factors such as BNI score, Vasograde, and cerebral edema, along with intraoperative parameters including bleeding and temporary clipping duration (>3 min), were significantly associated with outcomes. The derived AcoA prognostic score showed potential utility in predicting Glasgow Outcome Scale (GOS), ICU stay, and discharge outcomes at 6 months. These models leverage multimodal inputs, including imaging, clinical scores, and physiological data, to generate individualized prognostic estimates. However, similar to neuro-oncology applications, significant limitations persist (74).

## 5 The augmented surgeon: AI in the operating room

**Figure 4:**
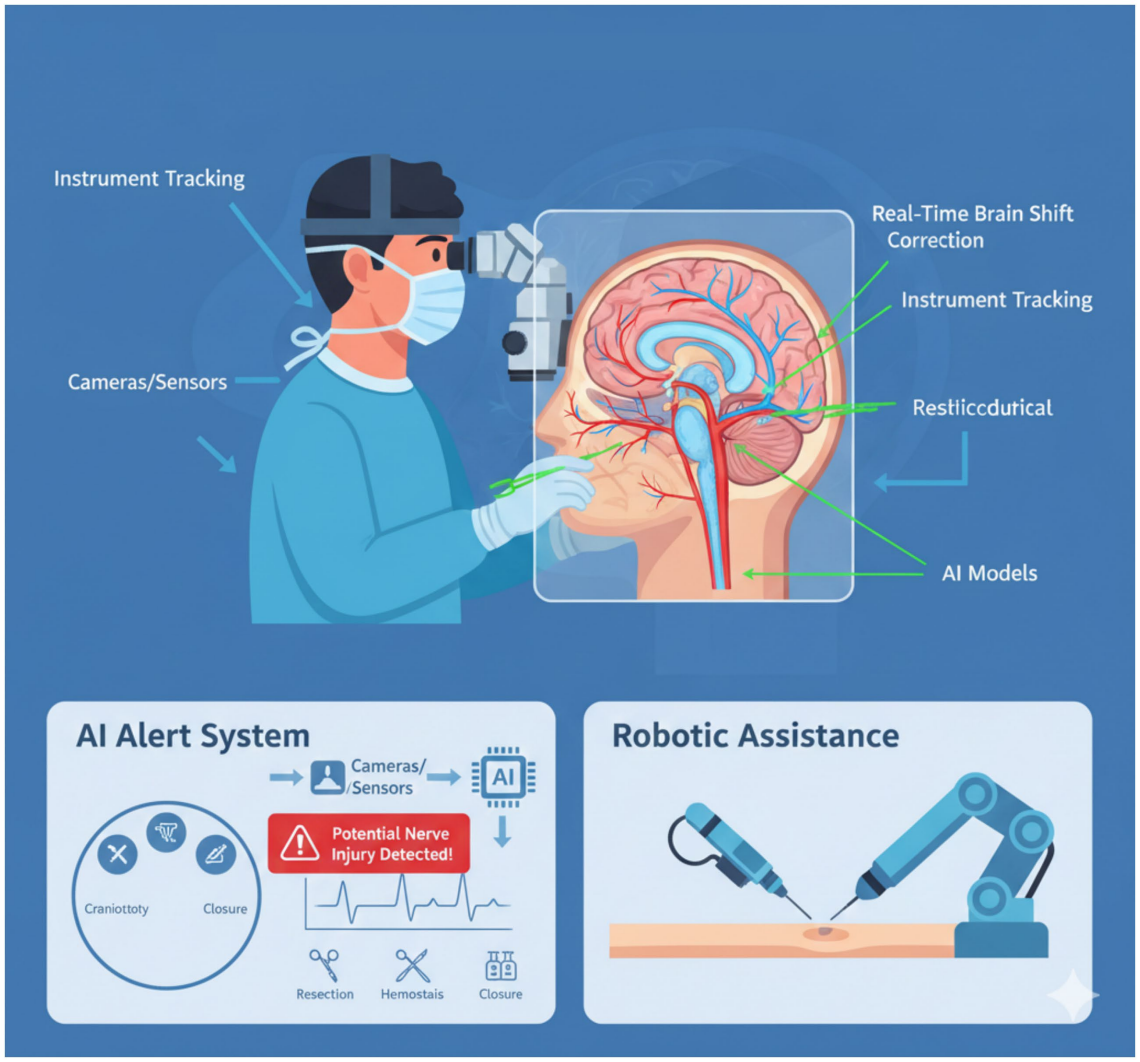
Intraoperative AI augmentation with augmented reality (AR) overlays.

The integration of AI into the neurosurgical operating room (OR) represents a paradigm shift from preoperative planning to dynamic intraoperative guidance. This evolution is giving rise to the concept of the “augmented surgeon,” where AI acts as a cognitive and perceptual coworker, enhancing human capabilities to achieve unprecedented levels of precision, safety, and efficacy (75, 76). By proceeding complicated, real-time data, AI systems are transforming the surgical environment into an interactive, information-rich ecosystem (Figure 4).

### 5.1 Surgical navigation and augmented reality overlays

Traditional surgical navigation systems, while invaluable, provide a disassociated view of the patient’s anatomy on a separate screen. AI is revolutionizing this by powering advanced augmented reality (AR) overlays that project critical anatomical and pathological data directly onto the surgeon’s field of view, either through a microscope ocular or AR head-mounted displays (77). These systems utilize deep learning algorithms to coregister preoperative MRI or CT scans with the live surgical scene in real-time, accounting for brain shift_ a significant limitation of conventional neuronavigation (78). The AI continuously refines the alignment, creating a dynamic and accurate fusion of the digital and physical worlds. This allows surgeons to see through tissue, visualizing hidden structures such as tumors, vasculature, and functional tracts directly superimposed on the surgical bed, thereby facilitating more complete tumor resections while minimizing collateral damage to adjacent brain areas.

### 5.2 Intraoperative monitoring and alertness: predicting and preventing neurological injury

AI enhances intraoperative monitoring (IOM) by advancing from threshold alarms to predictive alert systems. Leveraging machine learning on extensive datasets of electrophysiological signals like SSEPs, MEPs, and ECoG, these models identify complex patterns signaling potential neurological changes. For example, during surgeries such as aneurysm clipping, AI systems can predict motor deficits in real-time, enabling surgeons to make timely adjustments to prevent irreversible injury. This shift transforms IOM into a proactive safeguard for brain function (79).

### 5.3 Automated surgical phase recognition and skill assessment

Surgical education and quality control are being reshaped by AI’s ability to analyze the surgical workflow itself. Using computer vision techniques applied to video and kinematic data from the OR, AI models can automatically recognize and segment a procedure into its constituent phases (e.g., craniotomy, dural opening, tumor resection, closure) (80). This automated surgical phase recognition provides a structured log of the operation, which can streamline documentation, optimize OR resource management, and facilitate video-based review for training purposes. Furthermore, these same AI tools can be used for objective skill assessment. This data-driven approach promises to standardize neurosurgical training and accelerate the learning curve. With reported accuracies of 81–85% and weighted F1-scores ~0.83 in neurosurgical videos (e.g., vestibular schwannoma resection). While useful for workflow analysis and training, performance is phase-dependent and sensitive to video quality/variability across institutions (81). Deep learning models achieve 81–85% accuracy and F1-scores of approximately 0.83 in automated surgical phase recognition from neurosurgical video data.

### 5.4 The horizons of autonomous robotic assistance

The most forward-looking application of AI in the OR is the development of autonomous and semi-autonomous robotic systems (82). While current robotic platforms in neurosurgery are primarily telemanipulators under the direct control of the surgeon, the next generation incorporates AI to execute specific, well-defined tasks with superhuman precision (83). These systems are designed to carry out repetitive yet vital procedures like automated bone drilling in craniotomies, where AI provides feedback to halt at the inner cortical layer and reduce the risk of dural injury. In microsurgical anastomosis, AI-assisted robotic systems help stabilize tremors and perform sutures with sub-millimeter precision, alleviating surgeon fatigue. Importantly, the future of this technology is not aimed at replacing surgeons but fostering a collaborative relationship, where surgeons maintain strategic control while assigning specific technical tasks to robotic assistants, thus improving the precision and safety of surgical procedures (84). Table 2 summarizes the principal domains in which AI is currently influencing neurosurgical practice, spanning the diagnostic, intraoperative, prognostic, and postoperative stages of care.

**Table 2:** Major AI applications across the neurosurgical workflow.

Stage of care	Representative AI tasks	Techniques used	Clinical impact	Key references
Diagnostic / preoperative	Lesion detection, segmentation, radiomics, radiogenomics	CNNs, Radiomic feature extraction	Enhanced diagnostic accuracy, non-invasive genotype prediction	Zhou et al. (24) and Jian et al. (50)
Intraoperative guidance	Navigation, AR overlays, IOM prediction, phase recognition	Deep CNNs, Reinforcement Learning, Signal modeling	Improved precision, real-time risk alerts	Fida et al. (77) and Merghani et al. (79)
Prognostication	Survival prediction, complication forecasting	Ensemble ML, XGBoost, Multimodal integration	Personalized risk estimation, informed counseling	Della Pepa et al. (67) and Liu et al. (63)
Postoperative and long-term care	ICU monitoring, pain prediction, remote rehabilitation	RNNs, Predictive analytics, Federated learning	Reduced readmission, individualized therapy	Gabriel et al. (87) and Calderone et al. (90)

## 6 Postoperative care and chronic condition management

### 6.1 Predictive analysis for ICU stay and resource utilization

The immediate postoperative phase necessitates close monitoring and resource management. AI-powered predictive analytics are currently utilized to anticipate clinical trajectories, enabling more effective ICU administration. Machine learning models combine high-frequency, multi-modal data to estimate the probability of complications such as hemodynamic instability, respiratory failure, or the need for re-intubation. By accurately identifying patients who are at high risk for extended ICU stays, these models assist clinical teams in prioritizing resources and planning transfers to step-down units (85). Additionally, AI can develop ventilator weaning protocols and forecast vasopressor needs, resulting in more precise and timely clinical decisions that shorten ICU duration without compromising patient safety. This approach, driven by data, ensures that critical care resources are deployed with unmatched efficiency according to patient needs (86).

### 6.2 AI in pain management and opioid use prediction

Responsible pain management in neurosurgery is challenging due to risks like respiratory depression and dependence. AI models are being developed to customize analgesic regimens and predict individual opioid needs by analyzing preoperative factors (chronic pain history, genetics, psychological state), intraoperative data (opioid use, surgical regimen), and early postoperative assessments. These systems promote multimodal strategies to minimize opioid use while ensuring effective pain relief and categorize patients based on their risk of developing persistent opioid dependence, enabling targeted interventions such as referrals to pain management specialists (87).

### 6.3 Remote patient monitoring and early detection of deterioration

AI-driven remote patient monitoring (RPM) systems are critical for addressing post-discharge complications such as surgical site infections and hydrocephalus. These systems utilize data from portable devices and patient-reported outcomes to create individual baselines and detect deviations indicating clinical deterioration. For example, decreased mobility with self-reported headaches and sleep changes may signify risks. This early warning capability allows proactive interventions by healthcare providers, potentially preventing costly hospital readmissions (88).

### 6.4 Personalized rehabilitation protocols

Neurological and functional recovery in neurosurgical rehabilitation is evolving towards personalized regimens. For patients with spinal cord injuries, strokes, or tumor resections, AI algorithms assess initial deficits and performance data from robotic exoskeletons or virtual reality systems to dynamically adjust rehabilitation activities. This real-time adaptation enhances therapy complexity, type, and intensity, maximizing neuroplasticity and engagement for improved long-term functional outcomes (89, 90).

## 7 Navigating the challenges: the path to clinical implementation

A primary obstacle in the utilization of AI in neurosurgery is the “black box” dilemma, wherein the decision-making processes of complex algorithms, particularly deep learning models, are not easily understandable for human operators (91, 92). Neurological and functional recovery is a key goal of neurosurgical rehabilitation, shifting from standardized protocols to personalized regimens. For patients with spinal cord injuries, strokes, or tumor resections, AI algorithms evaluate initial neurological deficits and real-time performance data from robotic exoskeletons or virtual reality systems to adjust rehabilitation activities dynamically. This real-time adaptation of therapy complexity, type, and intensity maximizes neuroplasticity and engagement, ultimately optimizing rehabilitation efforts for better long-term functional outcomes (93).

In addition to interpretability challenges, the crucial issue of algorithmic bias and the need for health equity emerges. The effectiveness and applicability of any AI model are closely tied to the data it was trained on. If training datasets do not reflect the general population lacking diversity in ethnicity, gender, socioeconomic status, or geographic location the ensuring algorithms may reinforce or even worsen existing health inequalities (94). A model predominantly trained on data from a single demographic may experience reduced accuracy when applied to different patient groups, potentially resulting in misdiagnosis or inadequate treatment options for underserved populations. Addressing this risk necessitates the assembly of large, diverse, and multi-institutional datasets, along with thorough pre-deployment assessments for biased performance across various patient subgroups, to ensure that the benefits of AI in neurosurgery are fairly distributed (95).

A fundamental methodological limitation of this work is its narrative design, which lacks a standardized literature search protocol and formal quality appraisal, increasing the risk of selection bias. The heterogeneity in AI methodologies and reporting standards precludes quantitative synthesis, making conclusions about the superiority or readiness of specific AI tools cautious. Future systematic reviews and meta-analyses focusing on narrower subdomains are necessary for precise effect estimates and quality assessments. Additionally, limitations in the cited studies, such as retrospective designs and lack of external validation, temper enthusiasm for immediate clinical deployment.

A critical limitation in the translation AI from experimental settings to real-world neurosurgical practice is the persistent gap between algorithmic development and clinical implementation. While numerous models demonstrate high performance in retrospective or internally validated datasets, their deployment in clinical environments remains constrained by complex regulatory, ethical, and operational barriers. Regulatory frameworks such as those proposed by the U.S. Food and Drug Administration and the European Medicines Agency require rigorous validation, transparency, reproducibility, and continuous post-market surveillance, particularly for adaptive or continuously learning algorithms. However, many current AI systems lack standardized reporting, external validation, and prospective clinical trials, limiting their eligibility for regulatory approval and real-world adoption (96, 97).

Equally important is the necessity of XAI in high-stakes neurosurgical decision-making. The “black box” nature of deep learning models poses a significant barrier to clinician trust, accountability, and medico-legal acceptance. In safety-critical environments such as neurosurgery where decisions directly impact neurological function and survival clinicians must be able to interpret, verify, and justify AI-driven recommendations. XAI approaches, including feature attribution methods (e.g., saliency maps, SHAP values) and inherently interpretable models, are increasingly recognized as essential components for clinical integration (98). These methods not only enhance transparency but also facilitate error detection, bias identification, and alignment with clinical reasoning. Without such interpretability, even highly accurate models may remain unusable in practice due to lack of trust and regulatory non-compliance. Therefore, bridging the gap between AI innovation and clinical implementation requires a dual focus on robust regulatory pathways and the integration of explainability frameworks to ensure safe, ethical, and clinician-acceptable deployment (99).

## 8 Large language models (LLMs) in neurosurgery: emerging capabilities and challenges

Recent advances in Large Language Models (LLMs), particularly transformer-based architectures such as GPT-4 and Med-PaLM, are redefining the role of artificial intelligence in healthcare (100). Unlike traditional machine learning models that focus on structured or imaging data, LLMs are capable of processing and generating human-like text, enabling interaction with unstructured clinical information at scale. In neurosurgery, LLMs are increasingly being explored for clinical documentation, automated report generation, literature synthesis, and decision support. For example, LLMs can assist in drafting operative notes, summarizing complex patient histories, and extracting key findings from electronic health records (EHRs), thereby reducing administrative burden and improving workflow efficiency. Furthermore, integration with multimodal systems combining text, imaging, and structured data represents a significant advancement, allowing more holistic patient modeling and context-aware clinical insights (98, 101).

Beyond documentation, LLMs show promise in medical education and training, where they can simulate case-based discussions, provide real-time explanations of neurosurgical procedures, and support trainees in clinical reasoning. Their ability to rapidly synthesize large volumes of biomedical literature also positions them as valuable tools for evidence-based decision-making and research acceleration (102). However, despite these advancements, several limitations hinder their safe clinical deployment. A major concern is the phenomenon of hallucination, where LLMs generate plausible but incorrect or non-evidence-based information (103). In high-stakes environments like neurosurgery, inaccuracies can lead to serious clinical consequences. Significant barriers include data privacy issues, the need for domain-specific validation, biases in training data, and evolving regulatory frameworks like those from the U. S. FDA. Future research should prioritize fine-tuning generative AI systems on curated clinical datasets, utilize retrieval-augmented generation (RAG) techniques, and develop hybrid models merging symbolic reasoning with deep learning. Integrating large language models (LLMs) with explainable AI (XAI) frameworks and human-in-the-loop validation will enhance reliability and trust among clinicians. While LLMs offer transformative potential for medical AI, their application in neurosurgery demands careful validation and regulatory oversight to support clinical decision-making effectively (104).

## 9 Future directions and concluding remarks

**Figure 5:**
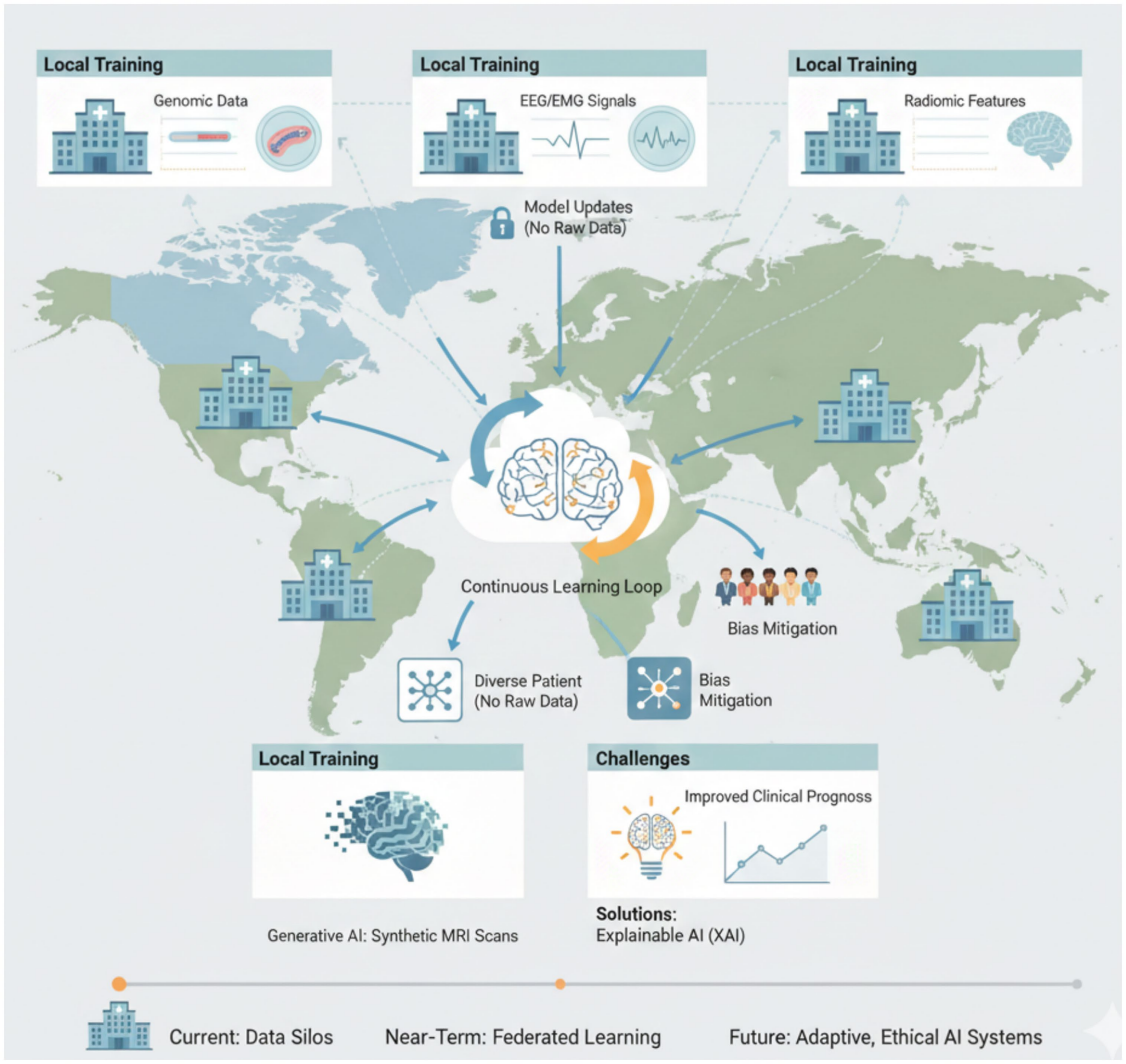
Federated learning ecosystem for ethical AI in neurosurgery.

The integration of artificial intelligence in neurosurgery is evolving towards advanced applications that go beyond diagnostics and predictive analytics, involving generative AI and continuous learning systems. Generative models are capable of producing realistic synthetic medical images, such as MRI and CT scans, which can illustrate diverse pathological conditions and anatomical variations. This innovation addresses significant issues by enhancing limited training datasets, offering limitless annotated examples to develop more effective and generalizable models while ensuring privacy. Additionally, it acts as a simulator for surgical training, enabling practice on rare or complex cases in a safe virtual setting. Furthermore, this technology fosters scientific advancement by formulating new hypotheses about disease progression and simulating potential treatment impacts (Figure 5).

In recent advancements, the field has introduced novel computational frameworks aimed at addressing the challenge of data siloing, with federated learning emerging as a transformative approach for multi-institutional collaboration (105). This method allows for the development of robust AI models without centralizing sensitive patient data. Rather than aggregating data in a central repository, the algorithm is sent directly to the data; models are trained locally at each participating institution, such as hospitals or research centers, with only anonymized model updates being shared to enhance a global model. This preserves patient privacy and institutional data sovereignty while utilizing diverse multi-center datasets to create equitable AI tools in neurosurgery. This convergence of technology is steering towards a future where a continuous learning healthcare system in neurosurgery is realized. In this model, AI evolves dynamically based on real-world clinical applications rather than being a static product following a single regulatory approval (106). As AI is implemented in operating rooms and clinics globally, its performance is continually assessed, and de-identified data is leveraged to iteratively refine its algorithms. This necessitates secure, ethical, and automated feedback pipelines for model updating, ensuring the AI system remains relevant and adapts to new trends and techniques, thereby enhancing its clinical value (107). Ultimately, this narrative review underscores that the evolution of AI in neurosurgery supports rather than replaces a surgeon’s expertise, serving as a critical augmentative tool (108). The future will be characterized by a synergistic relationship where AI handles complex data analysis, pattern recognition, and outcome prediction, allowing neurosurgeons to concentrate on higher-order decision-making and skillful procedure execution (109). By navigating challenges related to interpretability, bias, and integration; while harnessing the power of generative AI and federated learning, the neurosurgical community can direct this technology towards delivering superior, safer, and personalized patient care (110).

## 10 Conclusion

Artificial intelligence is rapidly reshaping neurosurgery, advancing the field from traditional scoring systems toward data-driven, precision care. Across diagnostic, intraoperative, and postoperative domains, AI enhances decision-making, prognostication, and workflow efficiency. Despite promising performance, challenges related to interpretability, bias, validation, and clinical integration remain significant. Addressing these barriers through rigorous validation, ethical frameworks, and explainable models will be essential. Ultimately, AI should be viewed as a collaborative tool that augments neurosurgical expertise, improving patient outcomes while preserving the central role of clinical judgment.
